# Opportunities and Recommendations for Improving Medication Safety: Understanding the Medication Management System in Primary Care Through an Abstraction Hierarchy

**DOI:** 10.2196/18103

**Published:** 2020-08-13

**Authors:** Andrew Baumgartner, Taylor Kunkes, Collin M Clark, Laura A Brady, Scott V Monte, Ranjit Singh, Robert G Wahler Jr, Huei-Yen Winnie Chen

**Affiliations:** 1 Department of Family Medicine University at Buffalo State University of New York Buffalo, NY United States; 2 Department of Industrial and Systems Engineering School of Engineering and Applied Sciences University at Buffalo Buffalo, NY United States; 3 Department of Pharmacy Practice University at Buffalo School of Pharmacy and Pharmaceutical Sciences State University of New York Buffalo, NY United States; 4 Department of Family Medicine Primary Care Research Institute State University of New York at Buffalo Buffalo, NY United States

**Keywords:** patient safety, polypharmacy, potentially inappropriate medications, primary care

## Abstract

**Background:**

Despite making great strides in improving the treatment of diseases, the minimization of unintended harm by medication therapy continues to be a major hurdle facing the health care system. Medication error and prescription of potentially inappropriate medications (PIMs) represent a prevalent source of harm to patients and are associated with increased rates of adverse events, hospitalizations, and increased health care costs. Attempts to improve medication management systems in primary care have had mixed results. Implementation of new interventions is difficult because of complex contextual factors within the health care system. Abstraction hierarchy (AH), the first step in cognitive work analysis (CWA), is used by human factors practitioners to describe complex sociotechnical systems. Although initially intended for the nuclear power domain and interface design, AH has been used successfully to aid the redesign of numerous health care systems such as the design of decision support tools, mobile patient monitoring apps, and a telephone triage system.

**Objective:**

This paper aims to refine our understanding of the primary care office in relation to a patient’s medication through the development of an AH. Emphasis was placed on the elements related to medication safety to provide guidance for the design of a safer medication management system in primary care.

**Methods:**

The AH development was guided by the methodology used by seminal CWA literature. It was initially developed by 2 authors and later fine-tuned by an expert panel of clinicians, social scientists, and a human factors engineer. It was subsequently refined until an agreement was reached. A means-ends analysis was performed and described for the nodes of interest. The model represents the primary care office space through functional purposes, values and priorities, function-related purposes, object-related processes, and physical objects.

**Results:**

This model depicts the medication management system at various levels of abstraction. The resulting components must be balanced and coordinated to provide medical treatment with limited health care resources. Understanding the physical and informational constraints on activities that occur in a primary care office depicted in the AH defines areas in which medication safety can be improved.

**Conclusions:**

Numerous means-ends relationships were identified and analyzed. These can be further evaluated depending on the specific needs of the user. Recommendations for optimizing a medication management system in a primary care facility were made. Individual practices can use AH for clinical redesign to improve prescribing and deprescribing practices.

## Introduction

### Background

Despite making great strides in improving the treatment of diseases, the minimization of iatrogenic harm continues to be a major hurdle. The process of treating illness often requires the use of medications with known adverse effects. The delicate balance of risk versus benefit is often complex and individualized, making it difficult to be addressed properly.

Medication error is a common cause of patient morbidity and mortality [1]**.** Medication safety encompasses preventing medication errors (eg, giving the wrong drug) as well as preventing harm associated with the intentional prescription of otherwise appropriate medications. Medication safety and thus the prevention of medical error require both the appropriate prescription of medication as well as their subsequent deprescription, a concept developed to address overprescribing [2]. 

Two important concepts in medication safety, especially significant in promoting deprescribing, are polypharmacy and potentially inappropriate medications (PIMs). The term polypharmacy is defined as the use of multiple medications concurrently by a single patient. These concepts assist with risk stratification for drug-drug interactions and adverse drug events. The exact number of medications varies among researchers and clinicians, but it is often considered to be 5 or more medications [2]. Medication is classified as a PIM if the risk of an adverse event is likely to outweigh its clinical benefit [3]. Patient-specific contextual factors, such as age and comorbidities, often drive the classification of drugs as PIMs that may otherwise be the standard of care. Both polypharmacy and PIMs pose an increased risk to patients, including increased rates of adverse events, hospitalizations, and increased costs [4-7]. 

Factors that contribute to medication safety are both at the system level, such as communication and system workflow, as well as the individual level, such as clinician education and experience. Screening tools, such as the Beers criteria, have been developed to assist in the identification of PIMs [3,8,9] in hopes of aiding providers in the identification of patients and medication that may require deprescribing actions. Tools that assist the provider in determining the appropriateness of the medication increase their willingness to deprescribe [10]. They have been applied in clinician-provided medication reviews, patient education and activation, and clinical decision support tools [11-16]. However, despite the availability of these resources, PIMs and polypharmacy continue to be a prevalent problem [17].

However, applying appropriate deprescribing concepts into clinical practice has proven to be not so straightforward. Individual clinicians report difficulty in addressing these issues due to barriers such as lack of time, lack of published clinical guidance of when and how to stop medications, and fear of poor disease outcomes related to stopping medications [18-20]. Meanwhile, system-based interventions designed to optimize the medication regimen of a patient population often have difficulty being implemented into existing, complicated health systems [21]. Implementation factors, such as the lack of pharmacist integration into the medical team, resource constraints, and individualized patient needs, limit the effectiveness of interventions. Traditional methods, therefore, may be insufficient to tackle such a complex problem, perhaps providing an explanation for the continued adverse outcomes associated with medications. 

In search of effective medication management strategies that support medication safety and deprescribing for everyday clinical practice, we propose to start by examining medication management in primary care as a system. To fully understand deprescribing, the management system as a whole can be evaluated with the long-term goal of promoting medication safety and removing PIMs. The primary care office represents a hub for clinicians and patients to exchange information and address medication issues on a regular basis. Exploring this hub in a systematic manner, informed by methods of human factors engineering, can help understand medication management and thus can be utilized to understand both barriers and facilitators of deprescribing.

### Objectives

As a first step toward understanding current practices in deprescribing at the primary care level, this paper presents a model of primary care medication management in the form of an Abstraction Hierarchy (AH), which seeks to describe the possible actions and constraints of work performed within a system [22]. By emphasizing the functional structure of a work system, rather than describing specific concrete situations, information requirements can be extracted from an AH that is independent of events and time, and thus can be used to design systems and interfaces that can handle novel and unexpected situations.

AH has been successfully applied in health care to understand domain constraints to facilitate the design of decision support tools [23], mobile patient monitoring apps [24], a telephone triage system [24], and various other workflow decision tools [23,25,26]. For example, Effken et al [23] modeled nurse’s decision support needs and constraints of the workplace on the design of computer interfaces to provide that support. Geӧrges et al [24] identified decision support needs required for monitoring patient vitals and communicating with other providers in the unit. This was used to create a mobile app for all nurses in the unit to check patient status and respond accordingly. These displays depict data at various levels of abstraction to visually represent the relationships between the required data components and complete tasks.

Unlike other analysis methods, such as task analysis (which aims to model the best set of actions to achieve a goal) or cognitive task analysis (a variant of task analysis to account for behavioral variability associated with different cognitive strategies), the AH emphasizes the system constraints and capabilities that operators act on (in contrast to task analysis of what operators do). These constraints and capabilities may then be used to explicitly identify information requirements for system design or redesign that can better support problem identification, efficient diagnosis, and effective problem solving. Information requirements extracted from an AH have been shown to differ from those generated from a hierarchical task analysis [27]. One significant difference between the 2 approaches is that task analysis is context dependent, such that actions and behaviors are derived for a specific goal or function. In contrast, an AH may be bounded by a context of use to help focus on the scope of the model, but is more widely applicable to the system across a broad range of situations [27]. For example, St-Maurice and Burns [28] developed an AH to model patient treatment. This analysis is bounded by activities within the clinician’s control, but does not exclude activities outside that specific workflow, such as processing the patient’s arrival to the office. 

The aim of this paper was to refine our understanding of the primary care office in relation to a patient’s medication through the development of an AH. In doing so, AH can be utilized as a guide for future studies, interventions, quality improvement as well as system and interface design. 

## Methods

### Initial Drafting

AH is a modeling tool that is a part of the work domain analysis, the first (out of 5) phase of cognitive work analysis (CWA), a human factors research approach for the analysis, design, and evaluation of work in complex sociotechnical systems. CWA was originally developed for use in the nuclear power industry to address the need for an optimized interface design of complex systems to prevent industrial accidents [29-31]. The methodology has since been adapted to a broad range of different sociotechnical systems, including health care.

Stanton et al [30] described a systematic way of approaching an AH. This methodology was utilized in our approach. Previous work, such as Read et al, Ashoori et al, Pigenot et al, and Xu et al [26,32-34], guided our analysis. This process included determining system boundaries, review, and consensus by a team of experts, followed by a detailed analysis. 

Determining the boundaries of the system, the first step in developing an AH requires capturing the appropriate amount of detail to describe the work taking place without populating the model with information irrelevant to the model’s objective [35]. Our model is intended to capture the medication management system of a typical primary care office. Primary care offices are central to all subdivisions of health care in the outpatient setting. Due to its central role in care coordination and disease prevention, the primary care office was an ideal system for addressing medication safety. We considered clinicians to be the *users* of the system in our analysis. 

Once the system of interest was determined, the AH was iteratively drafted in 2 alternating phases. The first phase included initial drafting of the components of the medication management system as *nodes* by 2 authors (TK and AB), one being a PhD student in human factors engineering and the other a resident physician in family medicine. Each node was subsequently added to an appropriate level of abstraction. Previous literature on AH methodology guided node creation and placement. 

Once each node was categorized at the appropriate level of abstraction, connections between nodes were made. Connections between nodes, also known as *means-ends* links, were then made. A means-ends link represents the connection of a node with nodes on a different level of abstraction. Each node was connected below its supporting nodes. In addition, each node was connected above its higher-level function or purpose. For example, the *Patient Assessment* node is connected above its end goals of *Patient-Centered Care* and *Appropriate Use of Medications*. The *Patient Assessment* node is simultaneously connected to the means by which it is accomplished below, which includes *Chart Review*, *History and Physical*, *Out-of-Office Communication Protocols*, and *Diagnostic Tests*. Thus, the resulting AH has each component of the system categorized into 5 levels of abstraction, with relationships between nodes being clearly demarcated. 

### Model Refinement

The second phase consisted of all authors coming together in an expert panel review of the draft AH. In addition to the student researchers, our team included 2 board-certified geriatric pharmacists, a clinical pharmacist, a family physician, a human factors engineering researcher, and social scientists. Collectively, the authors have extensive experience in education and research on PIMs, polypharmacy, deprescribing, and human factors analysis. Panel discussions provided key insights into a typical workflow of a primary care office, medication safety, ethical principles, and practical constraints in a clinical setting. Iterative discussions among the authors informed the subsequent revisions of the AH until a final model was agreed upon.

Once finalized, the AH was accepted to successfully represent the medication management system in a primary care office. The authors then carefully reviewed the AH with a focus on medication safety and deprescribing. On the basis of the AH and the existing CWA literature, recommendations for system design and improvement were drawn.

## Results

### Overview

**Figure 1:**
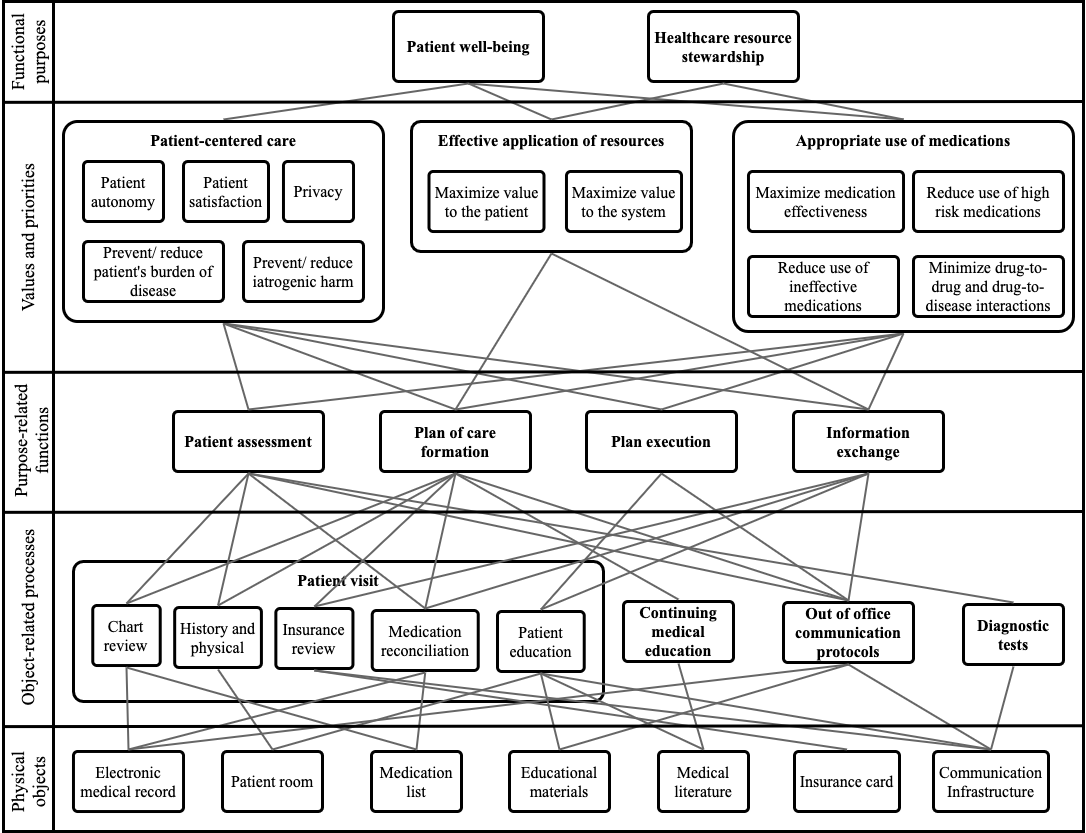
Abstraction Hierarchy of the medication management system in primary care.

The resulting AH graphically modeling the medication management system of a primary care office is depicted in Figure 1. The AH is a representation of the system at work. The model depicts each component of the medication management system in primary care at 5 different levels of abstraction. The top is the most abstract, and the bottom is the most concrete. In addition, it visually depicts the relationships between each component via *means-ends* links. With a model of the system at hand via the AH, the system can subsequently be optimized and improved. Although this AH can be utilized for a broad range of medication-related systems issues, we focus on deprescribing and medication safety.

### Functional Purpose

The functional purpose of the system is depicted at the top level of the AH. This level represents the overall goals of the system [30,32]. This level is also the most abstract. All lower levels and nodes function to support these top goals. In other words, the means by which these goals are accomplished are the connecting nodes below. When optimizing a system, these top-level nodes are used as the guiding end goals of the system.

A total of 2 functional purposes were identified in the AH: maximize *Patient Well-Being* and maintain appropriate *Healthcare Resource Stewardship*. Maximizing *Patient Well-Being* refers to maintaining or improving the patient’s current health status. *Healthcare Resource Stewardship*, as we define it, entails the effective and efficient mobilization of resources for the population. Resource allocation includes appropriate use of clinician’s time and pharmaceuticals when beneficial to the patient, as well as professional restraint when clinical benefits are unclear or unproven or are outweighed by real or potential harm.

The modeled system depicts achieving both goals simultaneously. Typically, these goals work in sync with each other for the benefit of everyone involved. Both can be sufficiently achieved in most scenarios. However, extreme cases may make achieving both these goals difficult, thus highlighting the constraints of the system. For example, some circumstances may necessitate prioritizing *Patient Well-Being* over *Healthcare Resource Stewardship*.

By understanding the relationships between the overall goals and the individual components of the medication management system in primary care, the system can be optimized to achieve these goals more effectively and efficiently.

### Values and Priorities

Values and priorities represent the way in which the system achieves its functional purpose [30,32]**.** They are the nodes most closely supporting the overall functional goals of the system. Each node at this level must be considered to support the above goals of the system. When optimizing a medication management system, these nodes must be utilized and balanced with each other to sufficiently support the functional purpose of the system.

A total of 3 categories of values and priorities were identified, which consist of *Patient-Centered Care*, *Effective Application of Resources*, and *Appropriate Use of Medications* [36]. These categories can be further classified into distinct yet similar components.

*Patient-Centered Care* refers to the clinician finding common ground with the patient to understand them and to better respond to their needs [37]. The central role of the patient is a key ethical principle used by clinicians to guide their work. The key role of *Patient-Centered Care* is consistent with existing literature. There are many documented benefits of patient-centered care for Patient Well-Being and Healthcare Resource Stewardship. This includes improved patient health, increased care efficiency, and reduction of unnecessary diagnostic testing and referrals.

If more specificity is required, *Patient-Centered Care* can be further classified into *Patient Autonomy*, *Satisfaction*, and *Privacy* [38]. Patient autonomy refers to the idea that patients should be involved in their care planning and be allowed to make their own informed decisions without undue influence [39]. Patient satisfaction encompasses a broad set of beliefs that cannot always be easily measured but must be evaluated in terms of the setting in which the patient is receiving care [40]. The importance of these nodes is consistent with existing literature, as satisfaction with primary care services has been shown to influence a patient’s health-related behaviors, such as compliance with medications [41].

In addition, *Patient-Centered Care* includes *Reducing the Patient’s Burden of Disease* and *Reducing Iatrogenic Harm*. These concepts refer to the worsening of a patient’s health status by the progression of their medical condition or through medical error [42]**.**

Another category at this level of abstraction is *Effective Application of Resources*. With limited health care resources and resources in high demand, any primary care office system must prioritize the effective application of resources to maximize value to their patient population. This includes minimizing out-of-pocket costs to the patient as well as minimizing costs for the health system at large. As expected, the impact of out-of-pocket expenses on health behavior is clearly observed by clinicians on a regular basis.

*Appropriate Use of Medications* is another value and priority of the medication management system in primary care. Although medications carry an inherent risk of iatrogenic harm, an astute clinician balances this risk with the medication’s benefits to improve patient well-being and effectively apply health care resources. To increase the specificity required to optimize the system, *Appropriate Use of Medications* can be classified into several nodes. This includes *Maximizing Medication Effectiveness* by using a pharmacologic intervention in specific patients who will benefit most from its effects. It also includes reducing the use of both high-risk medications and medications with minimal benefit. In addition, patient-specific risk factors such as drug-drug interactions and drug-disease interactions must be minimized. These underlying values and priorities can be observed regularly when, for example, clinicians reserve high-risk and expensive medications for patients most likely to benefit from the medication.

These values and priorities must be taken into consideration by the clinician while attempting to achieve the functional goals of the system. Although sometimes in sync with each other, certain circumstances may require the clinician to prioritize one above others. Understanding the competing values and priorities faced by the clinician is critical in optimizing medication management in primary care.

### Purpose-Related Functions

The next level of abstraction represents the purpose-related functions. This level is more concrete than those listed above and represents the work performed by the clinician. These are the general functions that need to be carried out to support the above goals of the system [30,32]. When designing or improving a system, understanding the relationship between these work functions and the rest of the system is critical. 

The purpose-related functions of the medication management system in a primary care office include *Patient Assessment*, *Plan-of-Care Formation*, *Plan Execution*, and *Information Exchange*.

*Patient Assessment* includes the evaluation of all patient-related information by the associated clinicians and the subsequent consolidation of information into a diagnosis. The patient expects that this assessment will inform the clinician of what action needs to be taken to maintain or improve their health, provide a basis for communication between them and their clinician, and allow them to voice health concerns that are most important to them [43]. Specific to our focus, accurate medication administration and evaluation of both desired clinical outcomes and patient reported negative effects are included in this function. The clinicians’ ability to assess the patient is one of their key work functions, and its importance to the overall system is intuitive. This is consistent with what is observed in the clinical setting, as electronic health record (EHR) systems frequently have a discrete section for the documentation of a clinician’s assessment. 

*Plan-of-Care Formation* involves the development of a plan to address the patient’s concerns and medical conditions revealed during the patient assessment. This plan may include items such as initiation, titration, or discontinuation of a pharmacologic agent, ordering a diagnostic test, regular monitoring, patient education, or referring to a specialist for further evaluation [44]. Although informed by the patient assessment, formulating the plan-of-care represents a distinct work function. When comparing this model to the real world, the plan of care is typically given its own section of the EHR. 

In the outpatient primary care setting, *Plan Execution* is a critical node that is often performed outside of the office and therefore left up to the patients or their caregiver. This includes medication adherence, going for laboratory evaluation, or making appointments with specialty services. This is commonly referred to as patient compliance and its importance in supporting Patient Well-Being and Healthcare Resource Stewardship is well known to clinicians. 

*Information Exchange* among all relevant stakeholders is another key work function performed in the primary care setting by clinicians. When comparing our model to a real-world primary care office, this refers to the patient education forms, sharing of medical records with other offices, electronic communication with other services such as laboratories, radiology departments, insurance companies, and pharmacies.

When observing the work of a clinician, it can be summarized into 1 of these 4 purpose-related functions. When optimizing the medication management system in a primary care office, understanding how these nodes relate to the rest of the system, including the overall functional purpose of the system, is critical. 

### Object-Related Processes

The fourth line in the AH represents object-related processes. These are the processes derived from physical objects, connecting physical objects to the higher functions of the system [30,32]. They are more specific and less abstract than the layer above. The processes at this level give purpose to physical objects in a way that serves the overall goals of the system; thus, understanding it is important for the optimization of the system.

There are numerous nodes at this level, as can be seen in Figure 1. For organizational purposes, many of these processes can be grouped together as a component of the *Patient Visit*. This includes *Chart Review*, *Medication Reconciliation*, *History and Physical*, *Insurance Review*, and *Patient Education*. These processes accurately reflect the components of a patient visit to a primary care office, reinforcing the model’s consistency to a real-world setting. Additional object-related processes include *Continuing Medical Education (CME)*, *Out-of-Office Communication Protocols*, and *Diagnostic Tests*. 

Similar to other levels of abstraction, these nodes must be integrated into the nodes above to serve the higher-level goals of the system. For example, a *Medication Reconciliation* is the process of creating an accurate, up-to-date representation of what medications the patient is currently taking. However, in order for it to be clinically useful, it must be integrated with the information discovered in the *Chart Review* and *History and Physical* to be clinically useful. This occurs during the higher-level process of *Patient Assessment*, where the clinician interprets and applies this information to maximize higher-level goals such as *Patient Well-Being*. This relationship highlights how the lower level, more concrete nodes across the system interact with higher-level ones to achieve the overall goal. 

Streamlining these processes with the goal of maximizing *Patient Well-Being* and *Healthcare Resource Stewardship* may appear initially difficult. However, the layers of abstraction between the object-related processes and the functional purposes facilitate the design of small components of the system to effectively support the overall goal of improved medication safety.

### Physical Objects

The fifth line and bottom of the AH represent the physical objects in the primary care office. At the bottom, these nodes are the most concrete of the AH. These are the resources and tools that clinicians use to make the system function [30,32]. However, without the processes previously listed, these objects have no relation to the overall goals of the system. 

Although these may vary slightly depending on the specific office being evaluated, many of these objects are universal. This includes the *Patient Room*, *Medication List*, *Educational Materials*, *Medical Literature*, *Medical Insurance Card*, *Communication Infrastructure*, *Office Space* and the *Electronic Medical Record (EMR)*.

An experienced clinician can note the difficulty of achieving the overall goals of the system, such as *Patient Well-Being* without certain resources, like a patient’s *Medication List*. However, for the medication list to be most useful, the clinician needs to perform a *Medication Reconciliation*, incorporate it into the *Patient Assessment* and *Plan of Care* while negotiating various priorities such as *Patient-Centered Care* and *Appropriate Use of Medications*. These connections emphasize the complex task imposed upon the clinician. By understanding the relationships and processes required by the system, the medication management system can be optimized with the clinician in mind.

Once developed, the AH can be used as a model for the medication management system in primary care. It visually depicts the numerous components in a primary care setting that needs to be sufficiently supported to achieve the overall goals of the system. This model can then be used to facilitate system optimization by guiding future quality improvement initiatives, research studies, and system and interface design.

## Discussion

### Principal Findings

By describing the system, it can then be analyzed for optimization. Our AH describes the medication management system in a primary care office. Further analysis reveals some general recommendations for building a primary care office designed with medication safety and deprescribing in mind. 

Interpreting an AH may not be straightforward for those not accustomed to such representations. Examining a particular node of interest and its associated links may lead to a more complete understanding of the functions and constraints surrounding an element within the medication management system. The first characteristic to note about a node is the level of abstraction within which it is embedded. Certain general recommendations, described below, can be provided to optimize that node based on this information alone. For example, value and priority are typically used as metrics to evaluate the functioning of the system. *Patient Satisfaction*, identified here as a value and priority, is already commonly used as a metric in clinical care due to its ability to provide feedback on the overall functioning of the system.

To evaluate the node of interest even further, a *means-ends* [32] analysis can be performed. The connections below connect to the supporting nodes, also known as the *means*. For the node of interest to work effectively, the supporting nodes must be designed to sufficiently support the node of interest. For example, *Patient Assessment* is supported by a *Chart Review*. Thus, the chart review process needs to be designed in such a way that it facilitates effective patient assessment to achieve the overall system goals. Some EHR systems clearly display medications that previously caused adverse events at the top of the patient’s chart, making *Chart Review* simpler and more streamlined. This quickly and effectively shares medication safety information for the clinician to incorporate into their assessment.

In addition, by looking at the connections above the node of interest, its goals can be seen. These are also referred to as the *end*. The node of interest must be designed with this function in mind, or else it is not relevant to the overall goal of the system. For example, *Patient Assessment* is connected above *Patient-Centered Care*. Therefore, patient assessment must be designed in a way that promotes patient-centered care. This includes asking questions related to how a disease is impacting their life (*Reduce Burden of Disease*), their input for what the patient would like to be done (*Patient Autonomy*), and asking about any possible medication side effects (*Reducing Iatrogenic Harm*). Without these types of questions, the patient assessment is limited in its ability to maximize patient well-being, which is the overall goal of the system. Many clinicians already ask questions like these, intuitively understanding their significance.

Although these examples reflect existing primary care practices, AH can also be used to suggest new practices that can be incorporated into quality improvement, research, and system and interface design. Some of the selected suggestions are explored in this analysis, leaving many more to be uncovered by further evaluations of the AH. The suggestions derived from the AH are inherently abstract but can be used to guide concrete improvements to the system. 

### Functional Purpose

The 2 functional purposes, *Patient Well-Being* and *Healthcare Resource Stewardship*, represent the high-level design objectives [30,32]. When altering system design, for any reason, it should be asked how the changes will end up impacting these 2 goals.

For example, when a quality improvement project is being proposed, it should include an evaluation of its anticipated impact on both *Patient Well-Being* as well as *Healthcare Resource Stewardship*. A given project may benefit both simultaneously or benefit one while harming the other. Any negative impact can be addressed and mitigated ahead of time, preventing a needless headache later.

### Values and Priorities

Values and priorities, representing the second highest goal of the system, reflect the overall functioning of the system. Monitoring these nodes provides insight into how the system is functioning [32]. By using these nodes as metrics, deficiencies in the system can be readily identified and addressed. Many of these are already in use as quality metrics in the primary care setting, such as patient satisfaction.

AH suggests that other metrics should be considered. The number of high-risk medications currently being used in a given patient population can reflect the effectiveness of the system. This metric is already used in research protocols to evaluate the effectiveness of interventions designed to deprescribe PIMs. Continuous monitoring of medication effectiveness may reveal the prevalence of ineffective and unnecessary medications in the primary care office. Keeping track of a patient’s out-of-pocket costs can be an evaluation of the financial strain that the current prescribing practices are placing on the patient. All of these measurements, and others visually depicted as values and priorities in the AH, may provide deeper insight into the functioning of the medication management system in primary care.

It is important to note that these values and priorities may be in conflict with one another, highlighting constraints on the system. The reduction of high-risk medications, as with the example above, must be balanced by reducing the burden of illness. This conflict has been reported in the real world during interviews with providers [18-20]. By identifying these conflicts ahead of time, they can be more easily addressed. Educating clinicians on how to navigate these conflicts may reduce the resulting burden on the system. For example, teaching clinicians to prioritize *Patient Autonomy* when making this difficult judgment call and thus morphing the conflict into an opportunity for the patient to take control of their care.

### Purpose-Related Functions

Purpose-related functions are the core work functions performed by clinicians. The design approach of task delegation, workflows, and user interfaces must be centered around these purpose-related functions to achieve the aforementioned values and priorities. Innovative and creative solutions are needed in the design of teams, user interfaces, task delegation, and workflow that effectively balance all the aforementioned values and priority nodes [32].

For example, *Patient Assessment* is completely delegated to the individual provider evaluating the patient. However, another model uses a more team-based approach. This may include delegating a component of this task to an in-house pharmacist dedicated to uncovering medication safety issues and making recommendations for deprescribing. This model has had some success in reducing the number of PIMs in a patient panel. In this situation, the pharmacist is utilizing the below *means* nodes such as *Chart Review*, *Medication Reconciliation*, and *History and Physical* to promote the higher-level *end* goals of the system, such as *Appropriate Use of Medications* and *Patient-Centered Care* [45]. The impact of this change can be monitored via the values/priorities as listed above, allowing for further iterations and fine-tuning for optimal results.

In addition, the importance of plan execution to support higher-level functions can be visually interpreted. Despite the primary care office being the coordinator of care, the actual execution of the care plan is often left up to the patient or their caregiver. This node may be the source of the deficiencies seen in the system. By supporting the *Plan Execution*, through the supporting nodes of *Patient Education* and *Out-of-Office Communication Protocols* the overall goals of the system may be better achieved. Examples of this could include a comprehensive patient education strategy as well as frequent follow-up after leaving the office.

#### Object-Related Processes

These nodes represent the tools and subprocesses that connect the physical objects to the higher functions and goals of the system. This level of abstraction can be optimized by the creation of novel and flexible tools that can be used to support the above *end* nodes. [32].

The AH shows how *Medication Reconciliation* is required to support *Patient Assessment*, *Plan-of-Care Formation*, and *Information Exchange*. Proper medication reconciliation, one that accurately documents the patient’s most up-to-date medication regimen, is therefore ripe for improvements that will have a large impact on the functioning of the system. As an example, one can envision an application that consolidates medication information from the patient, the pharmacy, and other prescribers and easily shares that information accurately with the primary care office. To be effective, this tool has to efficiently support the above *Patient Assessment*, *Plan-of-Care Formation*, and *Information Exchange* nodes. 

Many of the tools described in the medical literature are incorporated at this level. The creation of tools that are easy to use and fit efficiently within a workflow can be applied here to promote deprescribing. The Beers criteria, an existing screening tool, assists clinicians in formulating an assessment of the patient and has been used to assist with deprescribing. Another example of a hypothetical tool to promote deprescribing may be one for identifying and tracking previous adverse drug events (ADEs). Although a clinician may be able to find a previous ADE within the electronic medical record, this information is often not readily apparent. A tool that can perform this function is easy to use and fits into the existing workflow would support the patient assessment as well as information exchange.

#### Physical Objects

Physical objects, the lowest and most concrete level of the AH, are required to support all of the higher-level goals of the system. They are the means by which the clinician directly interacts with the system. For these objects to be most effective, they should be designed with flexibility in mind and offer clinicians choices that can be adapted for new and unforeseen circumstances [32]. Communication infrastructure is a clear example of the benefits of tool variability and flexibility in clinical practice. Most offices have patient-messaging systems, available phones, fax machines, and Health Insurance Portability and Accountability Act–compliant texting services among clinicians. The clinician has a variety of communication options available depending on the specific needs and circumstances called for by their situation.

EHRs are another opportunity for increased flexibility of the system. With the AH, it can be seen that EHR systems are required to support *Chart Review*. Therefore, a user interface that facilitates this function is key. To adapt to new and unforeseen circumstances, EHR systems that are flexible and allow for customization by the clinician are ideal. For example, a clinician may want to review previously prescribed medications and reasons for their discontinuation to inform their patient assessment and plan of care. An EHR system that makes this cumbersome may prevent the clinician from engaging in a review that may be informative and fruitful for the overall goals of the system. In other circumstances, however, this additional information may be too clustering and cumbersome for the task at hand. 

The same concepts of flexibility and variability can be applied to other nodes at this level. Patient educational materials should allow for variability based on patient health literacy, level of specificity, and preferred medium. For example, educational videos about medication side effects to monitor may be a more effective delivery tool for certain patients, whereas some patients wanting a deeper dive into the literature may prefer to be given direction to validated web-based resources.

### Limitations and Future Work

Our AH specifically evaluated the primary care medication management system from the clinicians’ perspective. Developing an AH from the patients’ perspective may yield a similar yet modified AH. The patients’ functional purposes are likely to be consistent with maximizing their well-being. Many values and priorities will likely overlap between a clinician’s perspective and a patient’s, including those related to patient-centered care and the reduction of harm. Greater differences between these models would be expected in the bottom 3 layers of the AH where these individuals would complete different tasks to achieve their goals.

Our AH is based on an expert panel of clinicians and human factors researchers, not direct observation. Although we attempted to include many different perspectives, the inclusion of more clinicians may have yielded a slightly different AH. In addition, all contributors to the model were based in Western New York. Higher-level purposes and priorities are expected to be consistent across all primary care practices throughout the United States; however, the processes and physical objects with which the clinician interacts may differ from site to site. Each site may modify how they complete these overarching goals based on the resources available and the population served by the primary care office.

Our future work includes an observational study, currently in planning, at a Western New York primary care clinic, and further observations at different types of primary care sites. Real-world data of clinical workflow and observed patient-clinician interactions would provide valuable data to help us better understand existing practices and barriers and to identify opportunities for appropriate prescribing and deprescribing opportunities. Such data will significantly add to the complex relationships modeled for medication management in general and deprescribing in particular.

### Conclusions

On the basis of the prevalence of PIMs, the current design of primary care work is inadequate in addressing the complex sociotechnical problems related to identifying and addressing PIMs. Deprescribing concepts, intended to improve medication safety, have been difficult to apply in the real-world setting. Our AH, depicted at a fairly general level for the medication management system in primary care, provides insights and suggestions for the optimization of the existing system. Some suggestions were explicitly mentioned in this paper, but numerous other interpretations can be made by those wishing to utilize this AH in the improvement of a specific primary care office.

Through the interpretation of this AH, human factors practitioners, administrators, and clinicians may identify and develop strategies to optimize the medication management systems and promote deprescribing in various primary care settings. By using the available means-ends relationships, the AH can be visually interpreted to determine which subsystems and processes need to be supported to accomplish the overall goals of the system.

Future studies, including our own efforts, should expand upon the subsequent steps in CWA to provide a more complete model of medication management work in the primary care setting.

## Abbreviations

ADE: adverse drug event
AH: abstraction hierarchy
CWA: cognitive work analysis
EHR: electronic health record
PIM: potentially inappropriate medication
